# Mortalidad causada por animales venenosos en Venezuela (2000-2009): nuevo patrón epidemiológico

**DOI:** 10.7705/biomedica.5561

**Published:** 2020-08-31

**Authors:** Leonardo De Sousa, Adolfo Borges, Enzo De Sousa-Insana, Aleikar Vásquez-Suárez

**Affiliations:** 1 Grupo de Investigación en Toxinología Aplicada y Animales Venenosos, Departamento de Ciencias Fisiológicas, Escuela de Ciencias de la Salud, Núcleo de Anzoátegui, Universidad de Oriente, Barcelona, Venezuela; 2 Laboratorio de Biología Molecular de Toxinas y Receptores, Instituto de Medicina Experimental, Facultad de Medicina, Universidad Central de Venezuela, Caracas, Venezuela; 3 Centro para el Desarrollo de la Investigación Científica, Asunción, Paraguay

**Keywords:** serpientes, himenópteros, escorpiones, mortalidad, epidemiología, Snakes, hymenopteran, scorpions, mortality, epidemiology

## Abstract

**Introducción:**

Los accidentes causados por animales venenosos ocurren con mucha frecuencia en comunidades pobres con acceso limitado a los servicios de salud. Se les consideran enfermedades desatendidas y son una de las causas importantes de morbimortalidad en varias naciones del mundo, incluida Venezuela.

**Objetivo:**

Evaluar la mortalidad por contacto traumático con animales venenosos (serie X20-X29) en Venezuela en el periodo de 2000 a 2009.

**Materiales y métodos:**

Los datos se obtuvieron de los anuarios de mortalidad del Ministerio de Salud.

**Resultados:**

Se registraron 759 decesos, la mayoría de ellos en el 2009. La primera causa fue la mordedura de serpientes (n=323; 42,6%), seguida por la picadura de himenópteros (n=170; 22,4%), la mordedura de centípedos (n=106; 14,0%) y la picadura de escorpiones (n=76; 10,0%). La mediana de la tasa de mortalidad general para el periodo fue de 0,285 fallecidos por 100.000 habitantes, en tanto que, por grupo específico, fue de 0,120 para ofidios, de 0,065 para himenópteros, de 0,035 para centípedos y de 0,025 para escorpiones.

**Conclusión:**

Al comparar estos datos con los antecedentes históricos, se evidenció la modificación del patrón de mortalidad en el país caracterizada por un aumento significativo de los decesos por centípedos, tercera causa de muerte, lo que reubica la picadura de escorpiones como la cuarta causa de mortalidad.

## Abstract (en)

**Introduction:**

Injuries by venomous animals frequently occur in impoverished communities with limited access to health services. They are considered neglected diseases that stand out as important causes of morbidity and mortality in various countries, including Venezuela.

**Objective:**

To assess mortalities resulting from contact with venomous animals in Venezuela from 2000 to 2009 (X20-X29 series).

**Materials and Methods:**

The data were obtained from the annual mortality records of the Venezuelan Ministry of Health.

**Results:**

From 2000-2009, 759 fatalities were recorded with the greatest number taking place in 2009. Snakebites (n=323; 42.6%) accounted for the largest percentage of envenomation-related deaths in that period, followed by hymenopteran stings (n=170; 22.4%), centipede bites (n=106; 14.0%), and scorpion stings (n=76; 10.0%). The median value of envenomation-related deaths per 100,000 inhabitants in 2009 was 0.285: 0.120 corresponded to snakebites, 0.065 to hymenopteran stings, 0.035 to centipede bites, and 0.025 to scorpion stings.

**Conclusions:**

Taking into account previous records of animal envenomations in Venezuela, we provided evidence for a shift in the pattern of mortality. Deaths due to centipede bites have increased, making it the third leading cause of envenomation-related mortality in Venezuela. Scorpionism, on the other hand, has declined to the fourth most common cause of fatal envenomations in the country.

La Clasificación Estadística Internacional de Enfermedades y Problemas de Salud en su décima revisión (CIE-10) constituye una herramienta fundamental para elaborar estadísticas de morbilidad y mortalidad para desarrollar políticas en salud colectiva (1). El capítulo XX de la CIE-10 engloba las causas externas de morbilidad y mortalidad (códigos V01 a Y98), y clasifica los acontecimientos ambientales y las circunstancias externas que resultan en traumatismos, envenenamientos y otros eventos adversos (1).

Entre las causas externas de morbilidad y mortalidad (códigos V01 a Y98), se encuentra el grupo de lesiones accidentales (códigos V01 a X59) que contiene un subgrupo denominado “otras causas externas de traumatismos accidentales” (códigos W00 a X59). Este subgrupo comprende dos categorías: a) la exposición a fuerzas mecánicas animadas (códigos W50 a W64) que incluye, entre otros, las mordeduras y los contactos traumáticos con animales; y b) los contactos traumáticos con animales y plantas venenosas (códigos X20 a X29) (1,2).

En su conjunto, los incidentes causados por animales (códigos W50 a W64 y X20 a X29) ocurren con mucha frecuencia en zonas rurales y en comunidades urbanas y suburbanas pobres con acceso limitado a los servicios de salud. Se clasifican como enfermedades desasistidas y se destacan como causa importante de morbimortalidad en varias naciones del mundo (3-11), incluida Venezuela (2,12-14).

En Venezuela, al igual que en varios países latinoamericanos, la información relacionada con el carácter epidemiológico y clínico de los accidentes causados por animales (venenosos y no venenosos) es insuficiente y es posible que también haya subregistro sanitario e, incluso, ausencia de registro por parte de las entidades municipales en algunas zonas geográficas (2). No obstante, se ha señalado que entre 1980 y 1999 fallecieron en el territorio venezolano 1.494 personas por contacto con distintos grupos zoológicos de vertebrados e invertebrados productores de venenos. El ofidismo representó la primera causa de mortalidad por envenenamiento (n=921 decesos; 61,7%), seguido por la picadura de himenópteros (abejas, avispas y hormigas) (n=310; 20,8%) y escorpiones (n=185; 12,4%), y la mordedura de centípedos (n=15; 1,0%) y arañas (n=5; 0,3%) (13). Este perfil fue prácticamente similar al registrado entre 1980 y 1990, con 877 decesos (14).

En el presente estudio se actualizó el perfil epidemiológico de la mortalidad por contacto con animales venenosos de la serie X20-X29 en Venezuela y se determinaron la magnitud y los indicadores de mortalidad para el decenio 2000-2009.

## Materiales y métodos

Se hizo un estudio descriptivo de tipo epidemiológico de corte transversal con diseño retrospectivo a partir de los datos de la vigilancia epidemiológica pasiva de la fuente secundaria sobre la mortalidad causada por animales venenosos registrados oficialmente para el periodo de 2000 a 2009 en Venezuela.

Los datos de mortalidad causada por animales venenosos registrados con los códigos de la serie X20 a X29 (CIE-10), se obtuvieron de los registros oficiales publicados en los “Anuarios de mortalidad” del Ministerio de Salud venezolano (http://www.mpps.gob.ve). Los códigos individuales correspondían a lesiones por contacto traumático con determinados animales o plantas venenosos, así: X20, serpientes y lagartos venenosos; X21, arañas venenosas; X22, escorpiones; X23, avispas, avispones y abejas; X24, ciempiés y milpiés venenosos; X25, otros artrópodos venenosos no especificados; X26, animales y plantas de origen marino; X27, otros animales venenosos; X28, plantas venenosas especificadas, y X29, animales y plantas no especificados (2,13). Se excluyó el código X28 por contener únicamente el contacto con plantas venenosas. Los códigos se organizaron en cinco grupos para evaluar la mortalidad: 1) serpientes (X20), 2) escorpiones (X22), 3) himenópteros (X23), 4) centípedos (X24) y 5) otros (X21, X25, X26, X27 y X29).

### Análisis estadístico

Para las causas que implicaron mortalidad, se determinaron las frecuencias y se calcularon las tasas de mortalidad anual (decesos por 100.000 habitantes) con base en la progresión de población suministrada por el Instituto Nacional de Estadísticas, INE (http//www.ine.gov.ve).

Para comparar las frecuencias, se utilizó el test de cálculo de diferencias entre proporciones (valor de z) con muestreo independiente. Se consideraron significativos los valores con p<0,05 (13). Para las tasas de mortalidad por causa específica, se calcularon las medianas como medidas de tendencia central para el decenio 2000-2009 y los quinquenios 2000-2004 y 2005-2009, según el método de Hodges y Lehmann, así como sus límites de confianza con 95% de certeza. En segundo término, las diferencias entre medianas se evaluaron mediante la prueba de análisis de varianza de Kruskal-Wallis al comparar más de dos medianas y la prueba de Mann-Whitney (Wilcoxon) en el caso de dos medianas con un nivel de significación de p<0,05 (2,12,15,16). Para ello, se utilizó el programa Est64 (11.55NF) para Windows 7 de 64 bits desarrollado por Carlos Sevcik del Laboratorio de Neurofarmacología Celular, Centro de Biofísica y Bioquímica (CBB), Instituto Venezolano de Investigaciones Científicas (IVIC), Venezuela (http://www.ivic.ve/cbb/labspa)

## Resultados

### Datos epidemiológicos generales

Se registraron 759 muertes por contacto traumático con animales venenosos en un lapso de 10 años (2000-2009) (en promedio 75,9 decesos por año) y la mayor frecuencia se observó en el 2009 (94 decesos; 12,4%) (cuadro 1).

**Cuadro 1:** Frecuencia anual de mortalidad causada por animales venenosos registrados oficialmente en la serie X20 a X29 en Venezuela, 2000-2009

**Año**	**Causa de mortalidad**												**Total**	
	**Serpientes** **X20**		**Himenópteros X23**			**Centípedos** **X24**			**Escorpiones X22**		**Otros** **X21, X25, X26,** **X27, X29**			
	**n**	**%**	**n**	**%**	**N**		**%**	**n**		**%**	**n**	**%**	**n**	**%**
2000	31	4,1	11	1,4	3		0,4	9		1,2	13	1,7	67	8,8
2001	45	5,9	22	2,9	9		1,2	10		1,3	5	0,7	91	12,0
2002	28	3,7	15	2,0	9		1,2	5		0,7	10	1,3	67	8,8
2003	34	4,5	12	1,6	3		0,4	6		0,8	12	1,6	67	8,8
2004	41	5,4	21	2,8	4		0,5	4		0,5	7	0,9	77	10,1
2005	45	5,9	18	2,4	9		1,2	6		0,8	10	1,3	88	11,6
2006	33	4,3	18	2,4	17		2,2	16		2,1	1	0,1	85	11,2
2007	23	3,0	16	2,1	13		1,7	4		0,5	6	0,8	62	8,2
2008	16	2,1	18	2,4	7		0,9	7		0,9	13	1,7	61	8,0
2009	27	3,6	19	2,5	32		4,2	9		1,2	7	0,9	94	12,4
Total	323^†^	42,6	170^‡^	22,4	106^¥^		14,0	76^£^		10,0	84	11,1	759	100

^†^ X20 contra X23: z=8,95; p<0,005

^‡^ X23 contra X24: z=4,28; p<0,005

^¥^ X24 contra X22: z=2,36; p<0,009

^£^ X22 contra “otros”: z=0,66; p>0,05

La primera causa de muerte por accidentes fue la mordedura de serpientes (serie X20) (n=323; 42,6%; z=8,95; p<0,005), seguida por la picadura de himenópteros (X23) (n=170; 22,4%; z=4,28; p<0,005), la mordedura de centípedos (X24) (n=106; 14,0%; z=2,36; p<0,009) y la picadura de escorpiones (X22) (n=76; 10,0%; z=0,66; p*>*0,05) (cuadro 1).

En el cuadro 2 se presenta la frecuencia de mortalidad por animales venenosos distribuida por quinquenios. Los resultados indicaron tres comportamientos: a) disminución significativa de la frecuencia de mortalidad por mordedura de serpientes en el segundo quinquenio (2005-2009) (n=144; 44,6%) comparado con el primero (2000-2004) (n=179; 55,4%; z=2,75; p<0,005); b) incremento significativo de la frecuencia para el grupo de los centípedos entre el primer quinquenio (n=28; 26,4%) y el segundo (n=78; 73,6%; z=6,87; p<0,005), y c) frecuencia similar para los himenópteros (z=0,86; p>0,05), escorpiones (z=1,29; p>0,05), otros animales (z=1,54; p>0,06), y el total de los envenenamiento (z=1,08; p>0,05) en los dos quinquenios.

**Cuadro 2:** Frecuencia de mortalidad causada por animales venenosos, serie X20 a X29, por quinquenios, Venezuela, 2000-2009

**Período**	**Serpientes**		**Himenópteros**		**Centípedos**		**Escorpiones**		**Otros**		**Total**	
	**n**	**%**	**n**	**%**	**n**	**%**	**n**	**%**	**n**	**%**	**n**	**%**
2000-2004	179^†^	55,4	81	47,6	28^‡^	26,4	34	44,7	47	55,9	369	48,6
2005-2009	144^†^	44,6	89	52,4	78^‡^	73,6	42	55,3	37	44,1	390	51,4
Total	323	100,0	170	100,0	106	100,0	76	100,0	84	100,0	759	100,0

^†^ z=2,75; p<0,005

^‡^ z=6,87; p<0,005

La frecuencia de mortalidad en cualquiera de los grupos taxonómicos fue mayor en hombres (n=607; 80,0%) que en mujeres (n=152; 20,0%) (cuadro 3). El grupo de edad con mayor mortalidad global causada por animales venenosos fue el de adultos jóvenes (20-44 años) (n=208; 27,4%), seguido por adultos mayores (>65 años) (n=182; 24,0%) y, en tercer lugar, adultos maduros (45-64 años) (n=160; 21,1 &) (cuadro 4). Al discriminar por grupos taxonómicos, la mayor cantidad de decesos por mordedura de serpientes se registró en adultos jóvenes (n=86; 26,7%), por picadura de himenópteros, en adultos mayores (n=85; 50,0%), por mordedura de centípedos, en adultos jóvenes (n=72; 67,9%) y por picadura de escorpiones, en escolares (n=30; 39,6%).

**Cuadro 3:** Frecuencia de mortalidad causada por animales venenosos, serie X20 a X29, según sexo, Venezuela, 2000-2009

**Causa de muerte**	**Sexo**				**Total**	
	**M**	**%**	**F**	**%**	**n**	**%**
Serpientes	243	32,0	80	10,5	323	42,6
Himenópteros	153	20,2	17	2,2	170	22,4
Centípedos	99	13,0	7	0,9	106	14,0
Escorpiones	48	6,3	28	3,7	76	10,0
Otros	64	8,4	20	2,6	84	11,1
Total	607	80,0	152	20,0	759	100

M: masculino; F: femenino

**Cuadro 4:** Frecuencia de mortalidad por animales venenosos, serie X20 a X29, según grupos de edad, Venezuela, 2000-2009

**Grupo de edad (años)^†^**	**Serpientes**		**Himenópteros**		**Centípedos**		**Escorpiones**		**Otros**		**Total**	
	**n**	**%**	**N**	**%**	**n**	**%**	**n**	**%**	**n**	**%**	**n**	**%**
Recién nacido/lactante (<2)	12	3,7	2	1,2	1	1,0	14	18,5	8	9,5	37	4,9
Preescolar (2-4)	10	3,0	5	3,0	0	0,0	24	31,7	1	1,2	40	5,3
Escolar (5-14)	41	12,7	6	3,5	0	0,0	30	39,6	7	8,3	84	11,1
Adolescente (15-19)	16	5,0	1	0,6	24	22,6	1	1,0	5	6,0	47	6,2
Adulto joven (20-44)	86	26,7	23	13,5	72	67,9	2	2,6	25	29,8	208	27,4
Adulto maduro (45-64)	83	25,7	48	28,2	8	7,5	0	0,0	21	25,0	160	21,1
Adulto mayor (>65)	74	22,9	85	50,0	1	1,0	5	6,6	17	20,2	182	24,0
?	1	0,3	0	0,0	0	0,0	0	0,0	0	0,0	1	0,1
Total	323	100	170	100	106	100	76	100	84	100	759	100

^†^ Los grupos de edad están distribuidos según las edades reflejadas en los anuarios de mortalidad.

### Indicadores de mortalidad

En el 2001 se registró la tasa de mortalidad anual más alta en el decenio, con 0,36 fallecidos por cada 100.000 habitantes para todos los grupos taxonómicos. En ese mismo año, las tasas de mortalidad por mordedura de serpientes y picadura de himenópteros registraron valores de 0,18 y 0,09, respectivamente. En el caso de los centípedos y escorpiones, la mayor tasa anual calculada fue de 0,06 para cada uno en el 2006 (cuadro 5). La mediana de la tasa de mortalidad para el decenio fue de 0,120 decesos por 100.000 habitantes para serpientes, 0,065 para himenópteros, 0,035 para centípedos y, en cuarto lugar, 0,025 para escorpiones.

**Cuadro 5:** Tasas de mortalidad anual por contacto con animales venenosos, serie X20 a X29, Venezuela, 2000-2009

**Año**	**Población**	**Serpientes**		**Himenópteros**		**Centípedos**		**Escorpiones**		**Otros**		**Total**	
		**n**	**TMA**	**n**	**TMA**	**n**	**TMA**	**n**	**TMA**	**n**	**TMA**	**n**	**TMA**
2000	24’069.837	31	0,13	11	0,05	3	0,01	9	0,04	13	0,05	67	0,28
2001	25’173.322	45	0,18	22	0,09	9	0,04	10	0,04	5	0,02	91	0,36
2002	25’537.781	28	0,11	15	0,06	9	0,04	5	0,02	10	0,04	67	0,26
2003	26’101.517	34	0,13	12	0,05	3	0,01	6	0,02	12	0,05	67	0,26
2004	26’564.394	41	0,15	21	0,08	4	0,02	4	0,02	7	0,03	77	0,29
Mediana 2000-2004		0,140 (0,120-0,155)		0,065 (0,050-0,080)		0,025 (0,010-0,040)		0,030 (0,020-0,040)		0,040 (0,025-0,050)		0,285 (0,260-0,310)	
2005	26’778.433	45	0,17	18	0,07	9	0,03	6	0,02	10	0,04	88	0,33
2006	27’466.601	33	0,12	18	0,07	17	0,06	16	0,06	1	0,004	85	0,31
2007	27’921.347	23	0,08	16	0,06	13	0,05	4	0,01	6	0,02	62	0,22
2008	28’374.877	16	0,06	18	0,06	7	0,02	7	0,02	13	0,05	61	0,21
2009	28’815.878	27	0,09	19	0,07	32	0,11	9	0,03	7	0,02	94	0,33
Mediana 2005-2009		0,100 (0,070-0,130)		0,065 (0,060-0,070)		0,050 (0,025-0,080)		0,025 (0,015-0,040)		0,027 (0,012-0,040)		0,275 (0,215-0,330)	
Mediana 2000-2009		0,120 (0,100-0,145)		0,065 (0,060-0,070)		0,035 (0,020-0,050)		0,025 (0,020-0,035)		0,035 (0,022-0,040)		0,285 (0,260-0,310)	

TMA: tasas de mortalidad anual

Entre paréntesis, límite de confianza de 95%

Aunque las diferencias entre las medianas de las tasas de mortalidad por grupo taxonómico no fueron significativas (p>0,05), evidenciaron la tendencia de tres comportamientos cuando se compararon ambos quinquenios: a) descenso de la mediana de tasa de mortalidad para serpientes, de 0,144 (2000-2004) a 0,100 (2005-2009) y para escorpiones, de 0,030 (2000-2004) a 0,025 (2005-2009); b) incremento para el grupo de centípedos, de 0,025 (2000-2004) a 0,050 (2005-2009), y c) estabilidad para los himenópteros, de 0,065 en los dos quinquenios (cuadro 5).

## Discusión

El territorio venezolano se caracteriza por su rica variedad de ecosistemas, lo que determina una gran diversidad animal con presencia de grupos zoológicos de importancia médica debido a su toxicidad (2,12-16), entre los que se encuentran los órdenes Anura, Araneae, Hymenoptera, Lepidoptera, Scolopendromorpha, Scorpaeniforme, Scorpiones y el suborden de Serpentes. Algunos de estos se localizan tanto en zonas urbanas como rurales y dan lugar a frecuentes interacciones que causan accidentes, los cuales pueden ser leves o tan graves que, incluso, pueden comprometer la vida del individuo afectado (14,17,18).

En Venezuela, la magnitud de la situación epidemiológica de los accidentes producidos por causas externas que implican animales venenosos apenas queda sugerida por los datos regionales aislados restringidos a pocos estados del país o circunscritos a registros hospitalarios de morbilidad (2,13). En el contexto nacional, los datos sobre el impacto en la salud colectiva son aún más escasos y están puntualizados en algunos indicadores, entre ellos, de mortalidad (19,20) y la morbilidad (12) específica por ofidismo, la mortalidad causada por distintos grupos de animales venenosos (13), o la morbilidad general por lesiones causadas por animales, que incluyen las mordeduras sospechosas de rabia (2).

En estas circunstancias y con base en los datos oficiales publicados por el Ministerio de Salud venezolano en los “Anuarios de mortalidad”, en este estudio se actualizaron y reconstruyeron el perfil epidemiológico y los indicadores de la mortalidad causada por animales venenosos en Venezuela, entre el 2000 y el 2009, y se compararon con los resultados previamente publicados por De Sousa, *et al*. (13) para el periodo de 1980 a 1999. Asimismo, se determinó la mortalidad específica para el ofidismo con respecto a la notificada por Benítez, *et al*. para el periodo de 1995 al 2002 (19) y por Parra, *et al*. para el periodo del 2003 al 2007 (20).

De Sousa, *et al*. (13) registraron, en 20 años (1980 a 1999), 1.494 fallecidos en el país por mordeduras o picaduras de animales venenosos: 789 en el decenio de 1980 a 1989 y 705 en el de 1990 a 1999. Los datos del presente estudio indicarían una frecuencia de 759 casos en el decenio de 2000 a 2009, cifra ligeramente superior a la del decenio precedente, lo que probablemente se relaciona con el incremento encontrado del número de decesos ocasionados por centípedos.

Benítez, *et al*. (19) registraron 266 muertes por ofidios en Venezuela entre 1995 y 2002, es decir, un promedio de 33 casos por año, fundamentalmente en hombres (70,7%) y con mayores tasas en los grupos de mayor edad. La tasa de mortalidad reportada por los autores se ubicó entre 0,1 muertes por 100.000 habitantes en 1997 y 0,2 en el 2002. Parra, *et al*. (20) notificaron 176 decesos por ofidismo entre los años 2003 y 2007, con un rango de tasa de mortalidad entre 0,08 y 0,17 muertes por 100.000. De Sousa, *et al*. (13) calcularon una tasa de mortalidad promedio anual para el ofidismo de 0,24 casos por cada 100.000 en un periodo de 20 años, entre 1980 y 1999: de 0,31 en el decenio de 1980 a 1989 y de 0,18 de 1990 a 1999. La mediana calculada en el presente estudio (aunque con un método estadístico distinto) señalaría un valor de 0,120 para el periodo 2000-2009, lo que evidenciaría una tendencia de disminución paulatina de este indicador en el ofidismo, similar al observado en los resultados obtenidos por De Sousa, *et al*. (13) y Parra, *et al*. (20).

En una revisión histórica de la mortalidad por mordedura de serpientes en Venezuela, se señaló que “[…] es notable la constante disminución de los indicadores tanto de mortalidad como de letalidad por ofidismo […]”, con valores promedio de mortalidad de 3,10 muertes por 100.000 habitantes y de letalidad de 32,4% (para 1947 y 1948) hasta llegar a una mortalidad de 0,15 muertes por 100.000 y una letalidad de 0,59% para el periodo 1996-1999 (13). La cifra calculada en en el presente estudio, de 0,120 fallecidos por 100.000, indica que se mantiene esta tendencia.

Sin lugar a dudas ello se debe a la progresiva eficacia en la conducta médica y terapéutica, y a la aplicación oportuna del tratamiento con antiveneno, que es más efectiva cuanto más precozmente se administre (12,13,15,18,21,22). También, se han mencionado como factores que explican el descenso de estos indicadores específicos el mejor acceso a los centros de atención médica, el aumento de la red de centros de salud con mayor distribución de antiveneno ofídico y el traslado oportuno de los pacientes a los establecimientos de atención sanitaria (13). Boadas, *et al*. (15) y Cornejo-Escobar, *et al*. (23) evidenciaron la importancia de la red ambulatoria en la recepción y tratamiento de los accidentes ofídicos. Se ha mencionado que este aspecto de la red ambulatoria “debe tomarse en cuenta, tanto para la distribución del antiveneno como para mejorar la calidad de atención en los centros asistenciales, evitando el traslado de los pacientes hacia sitios distantes a la localidad de ocurrencia del evento y, por lo tanto, disminuir la posibilidad de desarrollo de complicaciones, secuelas graves y/o eventos fatales” (13).

Chippaux (24) reportó un promedio por año de 5.700 accidentes ofídicos (incidencia de 18,87 casos por 100.000) en el país (periodo 1995-2012), con 32 decesos (mortalidad de 0,104 fallecidos por 100.000). Estos datos de mortalidad y morbilidad por ofidismo (32/5.700) indicarían una letalidad promedio anual de 0,56%, cifra cercana a la sugerida por De Sousa, *et al*. (13) de 0,59% para el periodo de 1996 a 1999. Además, el valor de la mortalidad previsto por Chippaux (24), de 0,104 fallecidos por 100.000 para el periodo 1995-2012, es cercano al de 0,120 fallecidos por 100.000 para el periodo 2000-2009 calculado en el presente estudio. En su conjunto, estos datos refuerzan la conclusión del descenso histórico de la mortalidad por ofidismo en Venezuela. Sin embargo, el autor (24) sugiere que la mortalidad por ofidismo en el territorio venezolano se ha mantenido constante, en tanto que la incidencia ha presentado un descenso drástico entre el 2006 y el 2012, lo que se asociaría con el deterioro de la recolección de los datos oficiales por parte de los entes que rigen la salud pública del país.

En segunda instancia, los resultados del presente estudio no descartan que la crisis social y migratoria que vive la población venezolana, con secuelas graves en su patrón de actividad económica, concurran en la disminución del riesgo de contacto con serpientes venenosas. Previamente se habían registrado 53.792 mordeduras de serpientes (5.976 casos, en promedio, por año) en un periodo de nueve años (periodo de 1996 a 2004), con una mayor incidencia en el 2004 (7.486 incidentes) y una mediana de la tasa de incidencia para el país de 24,46 accidentes por 100.000 en el lapso estudiado (12).

En cuanto a las otras causas de muerte por mordeduras o picaduras de animales venenosos, no hay indicadores regionales o por estado que reflejen la situación epidemiológica en el ámbito territorial nacional, excepto por la existencia de datos escasos y aislados de accidentes provocados por escorpiones en algunas zonas del país. En tal sentido, los territorios con mayor impacto en salud pública por picaduras de escorpiones se ubicaron en las regiones andina y nororiental de Venezuela (25). De Sousa, *et al.* señalaron que los estados de Mérida y Trujillo (región andina, al occidente del país) registraron en el periodo de 1996 a 2000 tasas de mortalidad promedio anuales de 0,50 y 0,28 por 100.000 habitantes, respectivamente, y los estados de Monagas y Sucre (región nororiental, al este de Venezuela) de 0,36 y 0,35 fallecidos por 100.000, respectivamente ((25)).Los indicadores de estos estados en ambas regiones fueron superiores a las del resto de las entidades federales y superiores, y a las registradas para todo el territorio venezolano de 0,08 fallecidos por 100.000 en el mismo periodo. Algunos autores han estimado una tasa de 0,04 muertes por 100.000 en Venezuela (26) y de 0,05 en Suramérica (7,27). Alves Araujo, *et al.* (28) calcularon tasas de mortalidad por escorpionismo de 0,29 y 0,20 por millón de habitantes (0,029 y 0,020 por 100.000) para Brasil en 1988 y 1989. Las registradas oficialmente en Venezuela en 1988 y 1989 (nueve y cinco decesos, respectivamente) determinaron proporcionalmente una mortalidad de 0,05 y 0,03 por 100.000 habitantes (14). Estos valores reflejaban un mayor impacto de la mortalidad en la salud pública en Venezuela que en Brasil para los mismos años. De Sousa, *et al*. (13) demostraron que la tasa de mortalidad promedio anual registrada en Venezuela por picadura de escorpiones en el periodo de 20 años entre 1980 y 1999 fue de 0,05 por 100.000.

La información de morbilidad por picadura de escorpiones para 1995 registrada oficialmente por la Dirección de Vigilancia Epidemiológica del Ministerio de Sanidad y Asistencia Social (29,30) indicaba una incidencia de 9,88 casos por 100.000 (847 accidentes) en el país, siendo los estados más afectados, en orden decreciente, Delta Amacuro (48,96 casos por 100.000; 56 incidentes), Monagas (23,63 por 100.000; 145 casos), Sucre (24,64; 177) y Mérida (19,4; 132). Para ese mismo año, otros autores (13) calcularon una letalidad muy alta por picadura de escorpiones, de 2,13% con base en una frecuencia de 18 decesos, y una tasa de mortalidad de 0,08 fallecidos por 100.000 al contrastar la frecuencia de muertes con la de casos (18 fallecidos/847 casos).

Se ha planteado que el accidente por picadura de escorpiones es un problema de salud pública más grave en algunas zonas del territorio que en otras (14,31). Los estudios publicados con información clínica o epidemiológica local, regional o nacional sobre otros grupos de invertebrados de la clase Arachnida, orden Araneae, han sido todavía más escasos (32-34).

Hay otros artrópodos que pueden producir envenenamientos y deben ser tomados en cuenta. Los ciempiés o escolopendras (centípedos) (clase Chilopoda) (35) vienen cobrando cada vez más importancia en la salud pública de Venezuela, tal como ha quedado evidenciado en la información de morbilidad publicada para algunas regiones (16,36-39) y los de mortalidad nacional para el periodo 1980-1999 (13). Tales antecedentes contrastados con los resultados de este estudio (periodo 2000-2009) indicarían el carácter emergente de este grupo taxonómico. Especialmente, los representantes del género *Scolopendra* (orden Scolopendromorpha) son depredadores terrestres muy eficientes y ampliamente distribuidos en las áreas tropicales del mundo (18,35).

En Venezuela, el contacto traumático con *Scolopendra gigantea*, denominado “escolopendrismo” (sic.), constituye un accidente agudo y muy doloroso que puede durar varias horas, con parestesias, eritema y edema local, limitación funcional y, raramente, desarrollo de ulceraciones o necrosis, pero según algunos autores, casi nunca se le considera un accidente serio o fatal (18,36-39). Los eventos, ocurridos a veces en brotes epidémicos, acontecen casi todos dentro del domicilio o en sus alrededores, fundamentalmente en la noche y en la estación seca del año (36,38,39), y han llegado a afectar incluso a neonatos (37). Sin embargo, es sorprendente la alta mortalidad asignada al grupo de los centípedos, lo que genera incertidumbre sobre la validez del diagnóstico taxonómico de estos casos y la posibilidad de que los accidentes mortales asociados con los centípedos hayan sido causados por otros grupos de artrópodos venenosos, especialmente los escorpiones.

En consecuencia, esta información, que resulta de las cifras consolidadas en la base de datos del sistema oficial de salud de Venezuela, debe valorarse críticamente, dada la confusión que podría suscitarse el asignar a los centípedos las muertes debidas a otros artrópodos erróneamente identificados en los niveles primarios de la atención en salud. Por ejemplo, algunos autores (12,15,16,40) han advertido sobre la existencia de fallas en los datos reales del accidente ofídico en el sistema de salud venezolano a nivel regional y nacional. En este sentido, se ha sugerido la evaluación “[…] del funcionamiento del flujo de transferencia de los datos con el fin de evitar la existencia de información disociada, con la consecuente subestimación o sobreestimación de la dimensión real del problema del ofidismo […]”. Esta misma situación podría verse reflejada en las cifras de muertes asignadas por error taxonómico a los centípedos y registradas en el presente estudio, lo que habría originado anormalidades en los números del sistema de salud en las regiones y el territorio nacional.

No obstante, incluso descartando la existencia de errores en la identificación de la categoría taxonómica, sería urgente evaluar si la mortalidad por centípedos es real y si se mantiene en el tiempo. Si tal evaluación confirma los datos, estaríamos frente a un interesante fenómeno toxinológico emergente en Venezuela asociado con envenenamientos graves con resultados fatales causados por ejemplares de la clase Chilopoda del orden Scolopendromorpha.

En este contexto, De Sousa, *et al*. (12,13) plantearon la necesidad de crear un sistema oficial de registro integrado de los datos (con flujo desde los ámbitos local, regional y nacional y entre ellos) que sea confiable, similar al *Sistema de Informação de Agravos de Notificação* (SINAN) de Brasil (2,12,13,15,16,40) con base en la notificación obligatoria del accidente ofídico a través de sistemas informáticos electrónicos de cobertura nacional que, además, debe necesariamente registrar otras condiciones clasificadas como enfermedades tropicales desatendidas causadas por animales venenosos (artrópodos o vertebrados, terrestres o acuáticos) y discriminadas según grupo taxonómico, con el fin de constituir un sistema funcional de vigilancia epidemiológica que abarque desde la localidad y el municipio donde ocurre el accidente hasta el espacio territorial regional y nacional.

Las causas de muerte por contacto traumático con animales venenosos (serie X20-X29) en este estudio ratifican que la primera es ocasionada por la mordedura de serpientes, seguida por la picadura de himenópteros. Sin embargo, en la tercera y cuarta posición los datos oficiales evidenciaron diferencias con los resultados publicados por De Sousa, *et al*. (13) para decenios anteriores. Las evidencias indicarían la existencia de una modificación de las causas específicas en el patrón epidemiológico histórico, pues el grupo de los centípedos –ubicado como la quinta causa de muerte en el periodo 1980-1990 (14) o como la cuarta en el de 1980 a 1999 (13)– pasó a ocupar la tercera posición (2000-2009), desplazando la picadura por escorpiones hacia el cuarto renglón de mortalidad.

Algunos autores (41-43) han planteado que el cambio climático global tiene efectos en las áreas de distribución geográfica y de comportamiento de los animales venenosos (escorpiones y serpientes), con la consecuente modificación de sus patrones epidemiológicos; es posible que este fenómeno afecte a los centípedos y esté potencialmente vinculado con la modificación de su dinámica epidemiológica en la interacción con el humano.

Estos resultados confirman que se mantuvo la tendencia en la disminución de la frecuencia de los fallecidos por ofidismo en Venezuela, patrón epidemiológico advertido ya por otros autores (13,20). Como nuevas dinámicas se observaron el incremento inesperado de la frecuencia de mortalidad debida al grupo de los centípedos y de otros animales venenosos, y la posible estabilidad de la frecuencia para los himenópteros y escorpiones. Se evidenció, asimismo, la importancia de la mortalidad causada por animales venenosos en Venezuela en el decenio de 2000 a 2009, lo que refleja la persistencia de este problema de salud colectiva en el territorio nacional y los indicios de modificación del patrón de mortalidad en el país con base en el aumento significativo de los decesos por mordedura de centípedos.
